# The shortfalls of vulnerability indexes for public health decision-making in the face of emergent crises: the case of COVID-19 vaccine uptake in Virginia

**DOI:** 10.3389/fpubh.2023.1042570

**Published:** 2023-05-03

**Authors:** Lydia Cleveland Sa, Erika Frydenlund

**Affiliations:** ^1^Storymodelers Lab, Graduate Program in International Studies, Virginia Modeling, Analysis, and Simulation Center, Old Dominion University, Norfolk, VA, United States; ^2^Storymodelers Lab, Virginia Modeling Analysis and Simulation Center, Old Dominion University, Suffolk, VA, United States

**Keywords:** COVID-19, vulnerability index, emergent crisis, public health policy, vaccine uptake

## Abstract

Equitable and effective vaccine uptake is a key issue in addressing COVID-19. To achieve this, we must comprehensively characterize the context-specific socio-behavioral *and* structural determinants of vaccine uptake. However, to quickly focus public health interventions, state agencies and planners often rely on already existing indexes of “vulnerability.” Many such “vulnerability indexes” exist and become benchmarks for targeting interventions in wide ranging scenarios, but they vary considerably in the factors and themes that they cover. Some are even uncritical of the use of the word “vulnerable,” which should take on different meanings in different contexts. The objective of this study is to compare four vulnerability indexes produced by private, federal, and state institutions to assess the application of these measures to the needs of the COVID-19 pandemic and other emergent crises. We focus on federal, state, and private industries’ vulnerability indexes for the Commonwealth of Virginia. Qualitative comparison is done by considering each index’s methodologies to see how and why they defined and measured “vulnerability.” We also quantitatively compare them using percent agreement and illustrate the overlaps in localities identified as among the most vulnerable on a choropleth map. Finally, we provide a short case study that explores vaccine uptake in the six localities that were identified by at least three indexes as most vulnerable, and six localities with very low vaccine coverage that were identified by two or fewer indexes as highly vulnerable. By comparing the methodologies and index (dis)agreements, we discuss the appropriateness of using pre-existing vulnerability indexes as a public health decision-making tool for emergent crises, using COVID-19 vaccine uptake as a case study. The inconsistencies reflected by these indexes show both the need for context-specific and time-sensitive data collection in public health and policy response, and a critical critique of measured “vulnerability.”

## Introduction

The COVID-19 pandemic has highlighted the inadequacy of applying already existing vulnerability indexes, which are rightfully limited in their scope of variables, to emergent crises, which carry their own vulnerability generating contexts and require context-specific application, measurement, and understanding of variables related to “vulnerability.” This is poignant in the ongoing push to increase vaccine coverage in the face of issues both around vaccine access and vaccine acceptance, where vaccine uptake and distribution highlight the dynamic and context specific nature of “vulnerability,” and how when addressing different aspects of disease burden and prevention, the idea of “vulnerability” may take on vastly different meanings, oftentimes not reflected in the pre-existing vulnerability indexes commonly used to design interventions aimed at addressing crisis.

Globally, the World Health Organization’s previously existing assessment of countries’ capacities to prevent and mitigate diseases with pandemic potential was found not to correlate with national COVID-19 health outcomes (1), demonstrating the way in which a previously existing vulnerability index may not be able to accurately capture or predict emerging vulnerability. At the country and regional level, the importance of the spatial distribution of vulnerability has become of increasing interest. In Kenya, for example, in response to COVID-19, researchers generated a social vulnerability index and epidemiological vulnerability index to be used in tandem and categorize geographical regions based on level of vulnerability—which was found to be heterogeneous across the country (2). In England, researchers created a socio-ecological COVID-19 vulnerability index (SEVI) and a Vaccine Hesitancy Index (VHI), and demonstrated that, when used together to identify intersections of these two indexes, the VHI complemented the SEVI and together could be used to effectively inform decision-making in response to COVID-19 (3). These studies demonstrating the intersections of vulnerability across indexes offer insight into the ways in which one such index alone may be limited. Vulnerability is both difficult to define and to predict, thus limiting standalone, preexisting vulnerability indexes in their ability to presuppose disease burden, and especially true of health-related behavior and decision-making.

This difficulty of prediction has similarly been true for vaccine uptake in the United States. A nationwide poll in March 2021, found that those most likely to not want to vaccinate against COVID-19 were not racial minorities as experts expected due to preconceived understandings of vaccine uptake vulnerability, but rather white Republican men (4)—not a population that commonly bears the brunt of health disparities. In Virginia, Native Americans had the highest percentage of vaccine coverage as of February 2022 (113.4% of eligible population having received at least one dose), whereas those classified as White or Black had the lowest (66.3 and 63.1%, respectively) (5). Across the U.S., the Center for Disease Control’s Social Vulnerability Index (SVI), was found to be associated with increased COVID-19 caseload early in the pandemic (May 2020), this association varied considerably across geographies (6)–indicating that while the traditionally recognized structural variables generating vulnerability are still important (such as access to transportation, language, minority status, and disability), there are other factors at play in the context of COVID-19.

Thus, one must consider that, in the context of emerging crises that continue to evolve quickly, historically used vulnerability indexes may not capture important, context-specific variables, such as social-cultural variables impacting vaccine acceptance, and therefore may not be able to properly prepare healthcare or other institutions for new and changing dimensions of vulnerability. Further, it is possible that in applying pre-conceived notions of vulnerability in the generation of data-capture mechanisms and related indexes, decision-makers are rendering potentially “vulnerable” communities invisible. This study examines existing vulnerability indexes in the Commonwealth of Virginia to explore (dis)agreement between indexes and discuss ways for moving forward to better define vulnerability for low vaccine uptake related to both vaccine acceptance and vaccine accessibility.

To this end, we explore some of the literature on vulnerability, including the conceptualization and quantification of vulnerability, and perform subsequent qualitative analyses of vulnerability indexes in the context of COVID-19 vaccine uptake. The analyses include a qualitative summary of agreement between four different state and federal vulnerability indexes, an examination of agreement between these indexes on which geographies in the state are most vulnerable, and an exploration of several cases across the state in terms of (dis)agreed vulnerability across indexes and current (at the time of writing) levels of COVID-19 vaccine uptake. We end with a discussion of possible implications in terms of vulnerability in the face of COVID-19 and other possible emergent crises, what this might mean in terms of rendering populations more vulnerable or invisible in the data, and some possible recommendations for policy and decision-making considerations in the context of vulnerability. The analysis closes with a discussion of limitations and future directions for more in-depth analysis.

### Disparities in vaccine uptake

The Commonwealth of Virginia, like the rest of the US, has been able to acquire enough vaccines such that that every eligible person could receive one. However, vaccines are going unused. As of February 2022, the Virginia Department of Health reported that it had used 67.2% of the vaccines received. By September 2022, 72.4% of the population was considered fully vaccinated (5). As vaccine uptake stalls, non-pharmaceutical intervention policies are lifted, and variants with higher infectivity emerge, the likelihood of a COVID-19 endemic future continues to rise. In the last quarter of 2022, there were consistently approximately 2 to 3,000 cases, 10 deaths, and 34 hospitalizations per 100,000 across Virginia (5).

Decades of public health research has shown that socio-behavioral and structural determinants play a critical role in vaccine uptake (7–12), yet disparities remain. To date, COVID-19 vaccination strategies have largely focused on equitable distribution. As COVID-19 has exacerbated existing inequities, and practitioners continue to push for high and equitable vaccine uptake to inform interventions, the question must be continually addressed: what socio-behavioral and structural determinants of health most influence COVID-19 vaccine uptake in Virginia?

While historic inequities have driven disparities in COVID-19 health outcomes, such as morbidity and mortality, differences in current vaccine uptake appear to *also* be largely driven by social, relational, and political phenomena. Vaccine rollout efforts that have prioritized equity are to be applauded in achieving high levels of vaccine coverage in many historically marginalized communities, such as the Native American communities in Virginia. However, as vaccine levels stall, practitioners must re-visit this question, and consider evolving outreach efforts to match the emerging trends in vaccine uptake and revisit the definition of who is “vulnerable” to not receiving the vaccine.

While much knowledge around vaccine confidence in the U.S is based on the National Health Interview Survey, studies indicate that factors associated with disparities in vaccine uptake may not be captured by this survey (13). Rightfully, equity was prioritized early-on in the COVID-19 vaccine rollout plans throughout the United States. Given the exacerbation of other health disparities in the context of COVID-19, there was fear that disparities in vaccine coverage may also be exacerbated. In fact, previous pandemics such as the H1N1 influenza pandemic saw disparity and inequity in vaccine uptake, with wide variance in vaccination rates between minority and majority groups (14, 15). Studies of routine vaccinations draw attention to similar health disparities (13, 16). Thus, the National Academies of Sciences, Engineering, and Medicine (NASEM) proposed a framework for equitable vaccine distribution, characterized by prioritizing benefit and doing no harm, prioritizing disadvantaged populations, and equitably considering difference or, in other words, avoiding a “color-blind” distribution and allocation schemes (17, 18).

In some contexts, this strategy has seen stellar success resulting in high vaccine coverage. However, vaccine uptake still lags, and the communities lagging are not necessarily those that were expected. Indeed, guidance highlighted the need to take social justice approaches (18). However, studies that have aimed to examine disparities in vaccine uptake associated with these determinants may reproduce the invisibilities by focusing on certain dimensions of vulnerability—such as urban versus rural—while perhaps disregarding others (16). For example, studies have been most often conducted online through Facebook (19) or crowdsourcing platforms (20), rendering potentially vulnerable communities, such as those with limited technology access, invisible.

Further, while historical studies have highlighted clear and ongoing disparities in vaccine uptake, some have noted the difference in difficult-to-reach and difficult-to-vaccinate populations, with a rise in difficult-to-vaccinate populations that do not necessarily fit the previous mold when it came to vulnerability in vaccine uptake. Equitable vaccination coverage goes beyond simply making the vaccine available, and includes dimensions such as gender, race, class, language, age, education, immigration status, religion or spirituality, self-perceived health and risk status, and geography (21, 22). It is therefore necessary to consider human factors, particularly the socio-behavioral and structural determinants of health. Vaccine uptake, the successful outcome of a vaccine campaign, is a *social* endeavor that goes beyond biotechnology and logistics to complex human dynamics (23). The Commonwealth of Virginia and Federal Government have been tracking COVID-19 vaccination data, and publishing some demographic variables related to vaccine uptake (percent overage of males versus females, for example), but we still know little about these human dynamics—the social and relational factors driving why people either choose not to vaccinate or are being left behind by vaccine campaigns.

These social factors, not covered in demographics used to develop vulnerability indexes or mathematical modeling approaches, must also account for the fact that people experiencing a pandemic can learn. People can, and do, change their minds. Early polls suggesting who was least willing to take a COVID-19 vaccine have become less informative as communities disproportionately impacted by the devastating impacts of the virus have been more receptive to the vaccine.

Vaccine uptake is particularly complex and context specific, depending not only on the time, place, and historic social determinants of health, but on additional social and relational factors, which are often extremely volatile and do not reflect the permanence associated with other, long-standing determinants of health. This volatility further highlights the need to continually revisit the question of what socio-behavioral and structural determinants of health are most influential on COVID-19 vaccine uptake in Virginia. In other words, which communities are vulnerable to low vaccine uptake? Thus, definitions of vulnerability in terms of vaccine uptake, and recommendations for an equitable approach to vaccine rollouts using such definitions were limited (1) by the timing of recommendations at the beginning of vaccine distribution planning and, as far as this study can tell, have not been updated and (2) by historic and ongoing invisibilities in the available data.

### Defining vulnerability for COVID-19 vaccine uptake

To examine how (in)appropriate various vulnerability indexes may be for application to target COVID-19 vaccine outreach, we must critically evaluate the mobilization and meaning of “vulnerability.” In this time and place, and for the COVID-19 vaccine, what and why is vulnerability?

“Vulnerability” has taken central stage in the public health discourse. However, critical discourse analysis has found that the use of the term is often vague or undefined, leaving open a wide spectrum of interpretation of who, what, and why vulnerability is (24). Throughout the COVID-19 pandemic, “vulnerability” and who is considered “vulnerable” has taken on different meanings. In the early stages of the pandemic, vulnerability was defined through biomedical characteristics, such as age, rather than social determinants (25). However, experts quickly noted the disproportionate impact of COVID-19 that historically marginalized persons (such as racial minorities) would and, for the large part, did bear (18, 26). These disparities are not new. They are reflective of health outcomes for other crises, such as H1N1 influenza and HIV/AIDS—and are a consistent finding across many long-standing and emergent public health concerns.

As these current disparities were reflective of historic disparities in health outcomes, previously generated vulnerability measures appeared to “fit.” Indeed, socially disadvantaged groups in the U.S, such as racialized minorities, have consistently carried disproportionate burdens of disease during pandemics. Experts indicate this is due to marginalized populations being “more vulnerable to illness, less able to protect themselves through preventive strategies, and more burdened than relatively privileged populations by public health response interventions” (27).

However, for vaccine uptake, there is perhaps more to vulnerability than shows up in pre-established equity indicators. Vulnerabilities compound and intersect across communities; racial or linguistic minorities may also live in rural areas with poor health access, lack trust in health institutions, and work in high-risk jobs. Additionally, many of these communities—rural, minority, and older adults have been hardest hit by the virus. They might have once been hesitant to vaccinate, but personal experiences with the virus can change someone’s perception of risk, as highlighted in reporting with community leaders in rural communities in western Virginia, attributing high vaccine uptake to the death of a prominent local political figure (28).

Vaccine confidence is one of the public health’s modern “wicked problems,” because it is the result of complex, nonlinear, and context-specific systems, and their relationship to individual decision-making processes. Like other aspects of public health, vaccine uptake has historically been linked to social determinants of health, such as minority status, language ability, transportation, income, and education (13, 15). For the COVID-19 vaccine, other factors such as religiosity have been found to play a role in vaccine hesitancy (22). Recognizing that, and identifying ways in which the determinants of health affect equity in vaccine uptake is key to advancing vaccination programs (21). Thus, in designing vaccine distribution schemes, these were the defining factors of vulnerability and where the focus was placed. As time goes on, we must re-assess the definition and mobilization of “vulnerability” for COVID-19 vaccine uptake. While scholars have attempted to do so using behavioral science approaches in various settings, including Poland and Canada (29, 30), this does not appear to be reflected in policy changes or changes to distribution schemes in the United States.

It is crucial to approach the matter with a health equity lens which critically evaluates the meaning of vulnerability, made possible by using a socio-behavioral and structural determinants approach. To understand the evolution of vulnerability as vaccine rollouts have continued, we must consider the most recent data and evidence. Notably, recent work has highlighted the “epidemiological mystery” of COVID-19 at the global scale, noting the importance of social and relational factors such as trust in a community’s ability to mitigate the impacts of the virus (1). Vaccine hesitancy, defined by Larson et al. as, “a state of indecision and uncertainty about vaccination before a decision is made to act (or not act),” was first identified at a global scale as a public health concern in 2010, and in the face of COVID-19 has become increasingly volatile (31). Particularly in the context of infectious diseases, both the temporal and spatial features of health behaviors such as vaccine hesitancy, coined “emotional epidemiology,” are of increasing importance For vaccine hesitancy, this is particularly true, as vaccine hesitant behaviors have been found to create pockets of unprotected communities through spatial clustering of the behavior, opening the door for (re)emergence of vaccine preventable morbidity and mortality (32). As vaccines became readily available and the pandemic moves toward endemic, we continue to ask whether the ideas of vulnerability which seemed to fit so well in the earlier stages of the pandemic continue to be appropriate in designing and implementing vaccine outreach strategies.

In considering this, it is important to critically assess dimensions of vaccine uptake, specifically in addition to any emerging context. A previously defined taxonomy proposed by Thomson et al. categorizes drivers of vaccine uptake as: *Access, Affordability, Awareness, Acceptance,* and *Activation* (33). Within vaccine *acceptance,* critical social perceptions of the vaccine and systems for delivery play a role. These are *confidence* (trust), *convenience* (access), and *complacency* (risk perception)– or the three domains of vaccine hesitancy as described by the World Health Organization’s Strategic Advisory Group of Experts (SAGE) on Immunization Working Group (34). *Access* and *Affordability* include geographical location and barriers such as transportation and financial limitations or considerations including time cost (33, 35). *Awareness* includes knowledge and information around the vaccine itself and how, where, and when to get the vaccine. Lastly, *activation* is meant to capture the effectiveness or influence of public health interventions driving vaccine uptake, such as ads, social media campaigns, or outreach (33). Importantly, the social determinants of health that directly influence vaccine uptake go far beyond race, and are characterized by marginalization and resulting vulnerabilities at the intersections of gender, race, sexual orientation, culture, geographic location, education, migration or documentation status, economic status, and context-specific structural determinants such as a history of redlining or Jim Crow era laws (9, 11, 21, 36), as well as contemporary social-political and relational issues. Thus, in defining ‘vulnerability’ for low vaccine uptake, Thomson’s 5As may be a starting framework by which to assess the appropriateness of vulnerability indicators.

## Methods

This analysis used existing open-source social vulnerability and health opportunity indexes created by private, public, and local entities. These include Surgo Venture’s COVID-19 Community Vulnerability Index (CCVI), the US Center for Disease Control’s Social Vulnerability Index (SVI), The US Federal Emergency Management Agency’s Social Vulnerability Index (SoVI), and the Virginia Department of Health’s Health Opportunity Index (HOI) (37–40). These measures are the benchmarks in their respective domains for calculating risk (or health opportunity in the case of the HOI) for populations with regard to health and/or disaster response. In other words, quantitatively defining vulnerability.

The CCVI is the only metric that was specifically developed to address COVID-19 vulnerability, taking into account many of the aspects of Thomson et al.’s “5 As” of vaccine uptake (33). Ultimately, the index is an adaptation of the CDC’s SVI, and thus has many similarities in the types of variables it covers, but adds four additional themes that are specific to COVID-19. The FEMA SoVI index is designed for vulnerability to natural hazards. These consider many of the same factors as the specific health vulnerability indexes, but it is likely that early vaccine hesitancy risks in the Commonwealth were assessed using these types of hazard indexes as a baseline. Notably, several of the indexes have not been updated in recent years, such as the FEMA SoVI (updated 2014); the CDC SVI’s most recent update was in 2020. Further, it is difficult to assess what is included in the measures of the variables in each index, because the publicly available information is limited. For example, FEMA notes that disabilities are important, but defines these as “medical disabilities” and is not clear how or what is included to measure medical disability. Finally, the Virginia HOI was specifically designed to identify health “vulnerability,” but in a generalized context not specific to COVID-19 or any particular disease. This index is the only one that scaled in such a way that low numbers were considered “bad” as in “low opportunity,” and high numbers were “good” (for “high opportunity for health”). For this reason, this index was used in its inverse for comparison with the others to determine relative health vulnerability.

The qualitative analysis component of this project is summarized in Table 1. This table captures what could be determined about the construction of each index based on those indexes’ publicly available methodology documents in terms of the number of variables the index captures regarding each theme.

**Table 1:** Qualitative comparison of four vulnerability indices.

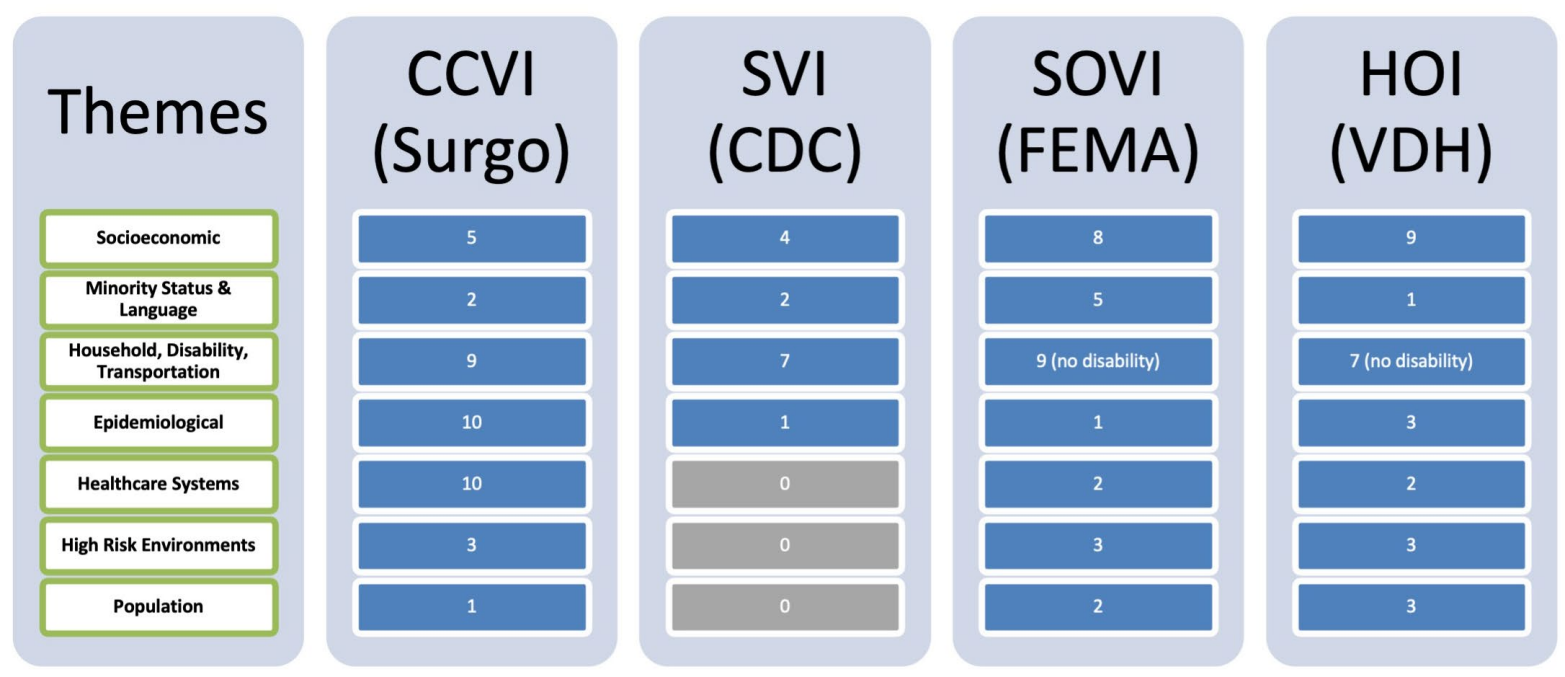

The variables used to create each index were categorized into major themes, attempting to condense them down into the fewest number of themes, and then qualitatively annotated to show variable coverage of those themes for comparison. Each index covers the identified themes of vulnerability to a varying degree of granularity and specificity. Examples of the types of variables that comprised each of the themes in the table include but are not limited to:

1. Socioeconomic: poverty levels, income, education, employment, job participation, economic inequality indexes
2. Minority Status and Language: population percentage of minority groups, English proficiency, and in the case of the HOI a “segregation index” that measures spatial segregation of ethnic groups
3. Household, Transportation, Disability: number of people in a household, types of houses (e.g., mobile homes, multi-unit structures), overcrowding, number of children in a household, single parent households, number of vehicles, and number of persons with disability
4. Epidemiological: population under five and over 65, cardiovascular conditions, respiratory conditions, immune-compromised, obesity rates, and diabetes rates
5. Healthcare Systems: health system capacity including number of hospital beds of different types, accessibility to healthcare providers, health preparedness for emergencies, and number of persons with health insurance
6. High-risk Environments: nursing homes, employment in the service industry, and employment in extractive industries (factories)
7. Population: population density, median age, percent female, and in/out migration

The “household” theme varied the most in respective index compositions. Transportation was most frequently categorized as whether or not the household had access to a vehicle, and was common across all indexes, while the Disability aspect was only covered in the CCVI and SVI as a census variable counting the number of people older than age five with a disability not in an institution. High-risk environments were specific to the COVID-19 context where a communicable respiratory virus affected people working in crowded conditions or institutions (37), as was the specific characterization of “epidemiological” factors in our qualitative grouping of the respective variables used to create each index. It should be noted here that the HOI is not grouped according to our thematic analysis. Using principal component analysis, the Virginia Department of Health grouped their over 30 composite variables into “profiles” of health opportunity that included: community environment, consumer opportunity, economic opportunity, and wellness disparity. To include this in our table, we used the component variables from the HOI regrouped along our themes to illustrate coverage of vulnerability-related measures.

**Figure 1:**
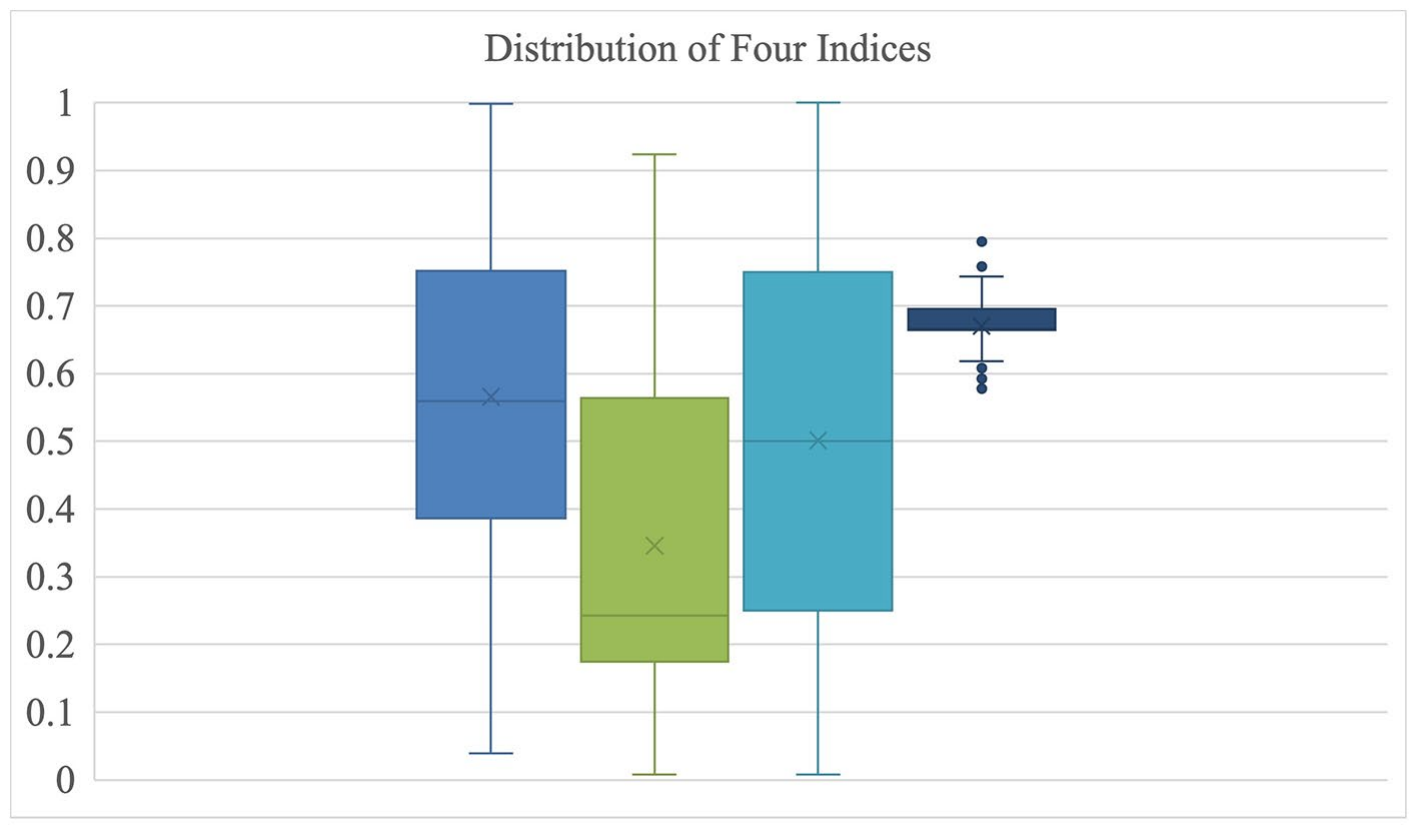
Distribution of four indexes. Left to right: Surgo CCVI; CDC SVI; FEMA SoVI; VDH HOI.

The quantitative component of this project consisted of a descriptive analysis examining the distribution of the quantitative measures of vulnerability for all the localities in the Commonwealth (Figure 1) and a percent agreement examination across indexes of the top 20% (i.e., the top 27 out of 133) of localities that each index named as being most vulnerable. For each index, we created a dichotomous variable where “1” represented a locality that it found to be in the most vulnerable 20% of all localities and “0” otherwise (i.e., 1 if the index ranked that locality in the top 27 most vulnerable in the Commonwealth). Indexes were compared pairwise (in sets of two) for percent agreement (see Table 2, below). In other words, we calculated the percentage of localities that each index agreed was “vulnerable” and “not vulnerable.” For reference, the list of localities in the top 20% for each index are listed in Supplementary Table S7 in the Supplementary material. In that summary table, there are 69 localities that at least one index determined was among the 20% most vulnerable in Virginia. Notably, none of the counties received agreement across all four indexes that it was among the most vulnerable.

**Table 2:** Pairwise percent agreement between indexes.

	FEMA SoVI	VDH HOI	CDC SVI
Surgo CCVI	0.7895	0.6391	0.6692
CDC SVI	0.6692	0.8045	
VDH HOI	0.6692		

Darker blues indicate higher percent agreement between indexes.

**Figure 2:**
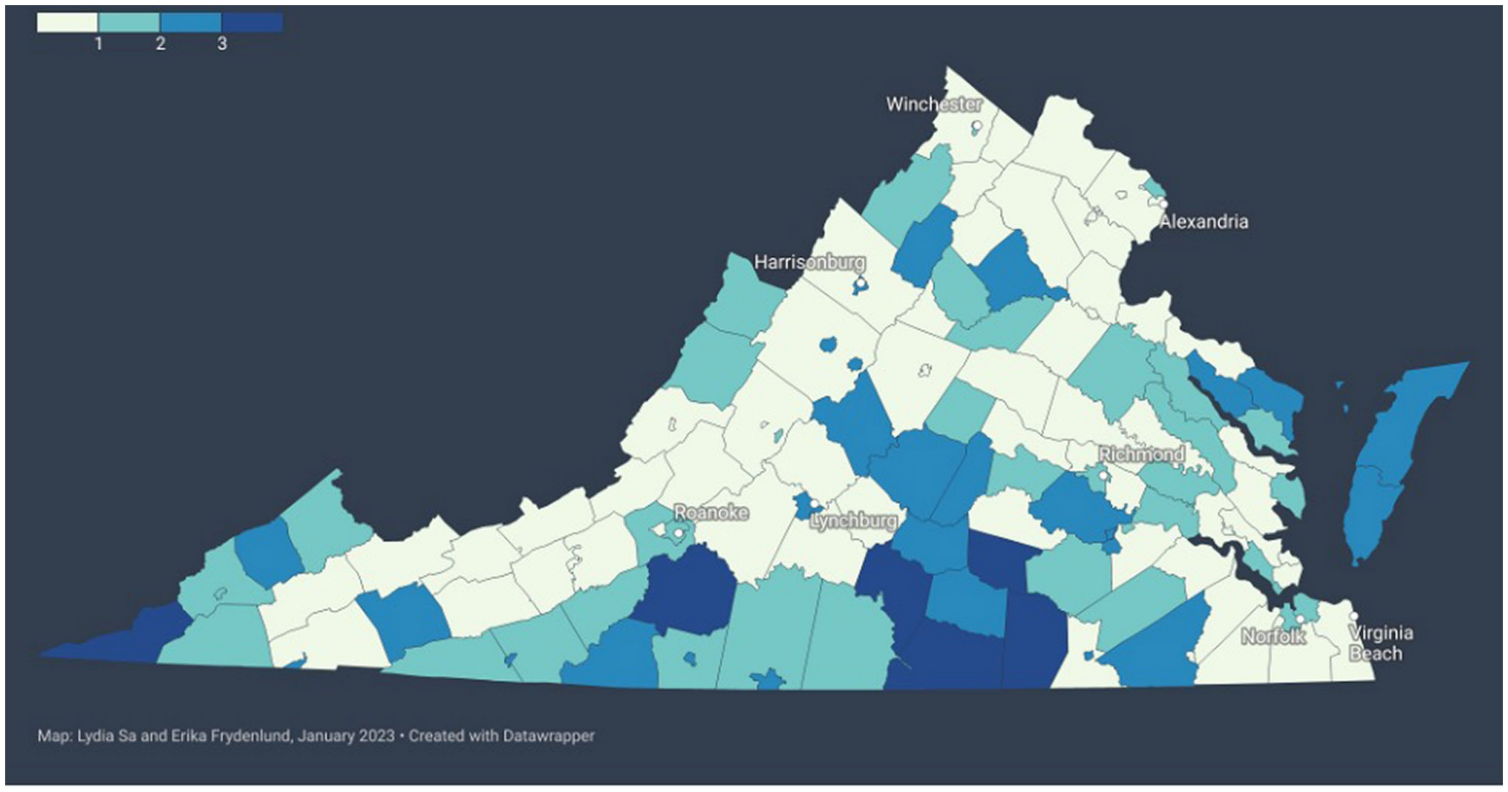
Comparing agreement between vulnerability indexes. Darker blue indicates higher number of indexes that agree in rating the locality as highly vulnerable (see legend in the top left).

The index data were further visually compared by constructing a choropleth map of the localities that had multiple indexes agreeing to label them as most vulnerable. This map-based visualization, shown in Figure 2 below, demonstrates that there was some agreement (but not consensus) about six highly vulnerable localities.

## Results

The vulnerability indexes represented here were each generated for their specific purposes, and thus vary considerably in the factors and themes that they cover. For example, the FEMA SOVI has a strong emphasis on household type, income, and minority because it is about recovery from natural disasters, whereas Surgo Venture’s CCVI has a strong emphasis on epidemiological variables since it focuses on COVID-19. Table 1 shows the qualitative comparison of the indexes by their publicly available methodology arranged by themes we determined by grouping the variables into categories. The value in each cell shows the number of variables in that index that covered the respective theme (rows).

Notably, disability was not well-covered by any of indexes, and two did not cover this area at all. Surgo Ventures’ CCVI index was the most robust at the surface, but the VDH HOI included a large number of variables that were aggregated into sub-indexes that were described in the VDH methodology documentation. Unsurprisingly, the CCVI, which was developed specifically for assessing COVID-19 vulnerability, included the largest number of epidemiological and healthcare system variables compared to the others, and these were specifically tailored to COVID-19. This is both a strength and a weakness of the index. On the one hand, the index captures the fact that vulnerability in terms of physical health for COVID-19 was increased for those with cardiovascular, respiratory, and immune-system conditions, as well as those diagnosed with obesity or diabetes. These similar underlying conditions may not necessarily add additional vulnerabilities in future public health crises and would thus need to be adjusted in the index to tailor to a new situation. The epidemiological consideration of the population of individuals aged 65 and over, however, was consistently present across all indexes as it creates vulnerabilities across a wider range of contexts, including natural disasters and broader health opportunities as well as the pandemic.

Figure 1, below, shows the distribution of values for each of the indexes. The way that the VDH HOI was constructed collapses the “health opportunity” of localities into a very tight distribution with several outliers in both the vulnerable and less vulnerable directions is notable. The CDC SVI, by contrast, is skewed toward classifying Virginia localities as less vulnerable in general.

In a two-by-two comparison of each index’s identification of the most vulnerable localities, we conducted a pairwise calculation of the percent agreement (see Table 2). While they are using, in some cases, vastly different sets of vulnerability metrics, the different indexes are generally at about 70% agreement when identifying the Commonwealth’s most vulnerable localities. The VDH HOI and CDC SVI have the highest percent agreement, at 80%, whereas the VDH HOI and Surgo Ventures CCVI have the lowest percent agreement, at 64% (see Table 2).

Notably, there is never a consensus across all four indexes that a particular locality is one of the most vulnerable (top 20%). Out of the 69 localities that at least one index considered most vulnerable, there were six instances where three indexes agreed, 27 where two indexes agreed, and 36 where only one index identified the locality as most vulnerable.

Figure 2, below, illustrates this point of agreement between indexes visually using a choropleth map of the Commonwealth of Virginia. In this representation, color indicates the number of indexes that agree on including that locality as one of the most vulnerable (top 20%) in the state. The darker the blue, the more indexes agree that it is a vulnerable locality. As shown in Figure 2, large swaths of the state are left without agreement between vulnerability indexes.

This disagreement includes some of the most populous regions of the Commonwealth, such as counties in Northern Virginia and Hampton Roads. Additionally, as of September 2022, even where there is agreement, such as on the Eastern Shore of Virginia, there are some of the highest proportions of persons fully vaccinated and with at least one booster or additional dose of the COVID-19 vaccine (65.1% + and 45.1% + respectively), whereas some other localities with high index agreements of vulnerability do also have relatively low vaccine coverage (50.1% + and 25.1% + respectively) (5).

We can further interrogate the consensus of these vulnerability indexes by looking at the six counties that at least three indexes agreed were among the 20% most vulnerable. Table 3 summarizes the percentage of eligible adults fully vaccinated in each of these six counties and which index identified it as “vulnerable.” Most of these vulnerable counties (apart from Lee County and Franklin County) had vaccination rates over 60% as of January 2023, which is higher than some counties (see Table 4), but lower than others in the state. At this time, the countries in Virginia with the highest proportions of fully vaccinated adults range between 80 and 90% coverage (5).

**Table 3:** Percentage of eligible adult population fully vaccinated in six counties that at least three indexes agreed were “vulnerable.”

	Adult (%)	Surgo CCVI	CDC SVI	FEMA SoVI	VDH HOI
BRUNSWICK COUNTY	64.8	Yes	Yes	No	Yes
CHARLOTTE COUNTY	63.3	No	Yes	Yes	Yes
FRANKLIN COUNTY	57.3	No	Yes	Yes	Yes
LEE COUNTY	46.7	Yes	Yes	No	Yes
MECKLENBURG COUNTY	65.5	Yes	Yes	Yes	No
NOTTOWAY COUNTY	65.7	Yes	No	Yes	Yes

**Table 4:** Percentage of eligible population fully vaccinated in six counties that had among the lowest vaccination rates in Virginia.

	Adult (%)	Surgo CCVI	CDC SVI	FEMA SoVI	VDH HOI
CARROLL	48.0	No	No	No	Yes
CRAIG	46.7	No	No	No	No
LYNCHBURG	54.0	Yes	No	Yes	No
PATRICK	51.3	No	Yes	No	Yes
PRINCE EDWARD	49.8	No	Yes	No	Yes
TAZEWELL	51.8	No	No	No	No

By contrast, Table 4 shows six of the counties with the lowest vaccination rates that did not have a majority of indexes identifying them as “vulnerable.” In this table, we can see that two counties with very low vaccination rates did not have one single index identify them as among the 20% most vulnerable (Craig County and Tazewell County). These counties saw low vaccine uptake across all identified race categories. In Craig County, for instance, just 5.4% of the Black adult population eligible for vaccines completed the full vaccination schedule, and in Tazewell County this number was just 20.7% (see Supplementary Tables S5, S6 in the Supplemental material). Carroll County was only identified by the VDH Health Opportunity Index as being vulnerable, despite an eligible adult vaccination rate at just 48%.

## Discussion

In summary, there is no total agreement on a single locality being one of the most vulnerable in the Commonwealth, and the highest level of percent agreement across indexes was 80% between the VDH HOI and CDC SVI, but the HOI is also in the lowest agreement across indexes, with a 64% agreement with the Surgo Ventures CCVI. The CCVI, based on the SVI, is the only index examined which was tailored to the COVID-19 response—specifically including and weighting variables important in the context of a respiratory infectious disease. Thus, it is concerning that this index reflects the lowest percent agreement measured, and that low percent agreement happens to be with the HOI—the tool generated by the Virginia Department of Health, and the index most likely used by the same Department of Health to inform decision- and policy-making in response to the pandemic.

The qualitative analysis indicates that, while the CCVI includes a considerable number of variables specifically related to the healthcare system and its capacity, the HOI includes relatively little. This could perhaps present a significant oversight if the HOI was used to inform decision-making around strengthening or providing support to healthcare systems in response to the pandemic. However, disagreement between indexes is not the only issue at hand.

As we explored in the introduction and background of this paper, vulnerability is both a structural and social phenomenon. While barriers to accessing healthcare in the United States—such as lack of access to transportation, low English proficiency, and limited or lack of health insurance—are certainly drivers of vulnerability and both generate and exacerbate health disparities, the spatial distribution of social phenomena also contributing to vulnerability and disparities is becoming more widely recognized as being of significance.

When it comes to the structural barriers contributing to vulnerability, the indexes are in relative agreement (see Table 1). However, given the context-specific nature of vulnerability, this index agreement should be as critically interrogated and questioned as the index disagreement. As noted in the results, for example, disability was not well-covered by any of indexes, and two did not cover this area at all. This appears to be counterintuitive, as disability, particularly physical disability, has been linked with vulnerability to respiratory illness and increased risk of complications and hospitalizations (41). The Pan American Health Organization (PAHO) notes that physical limitations related to disability are often a barrier to accessing health services, and that, in the context of COVID-19, persons with disabilities are less likely to be vaccinated against the virus (42). This lack of consideration of disability related to health vulnerability may be reflective of broader social issues around disability, such as the “disability-death” discourse—language that equates disability to death, essentially normalizing disparities suffered by those with disability—that has been recognized as problematic (43). This commonality across indexes of vulnerability to exclude or minimize disability effectively renders invisible those with disabilities and may contribute to further exacerbation of both vulnerability and health disparities.

Perhaps the most notable social phenomena in the context of the COVID-19 pandemic are vaccine hesitancy and uptake. Applying Thomson *et. al.*’s 5A’s as a framework for understanding drivers, and therefore vulnerability, for vaccine uptake, we may qualitatively assess whether the indexes available are appropriate. Recall, the 5A’s are *Access, Affordability, Awareness, Acceptance,* and *Activation.* The CDC SVI includes four primary dimensions of vulnerability: socioeconomic status, household composition and disability, minority status and language, and housing type and transportation. Broadly, these fit within the first two A’s: *access* and *affordability*. However, the SVI does not appear to address the remaining three dimensions of vaccine uptake: awareness, acceptance, and activation. Thus, while vaccine hesitancy and decision-making around whether to get the vaccine or not is largely not captured by the already existing vulnerability indexes. Perhaps due to the context-specific and complex nature of the issue, the spatial distribution of this ‘emotional epidemiology’ (31) is of increasing importance and should not be disregarded.

This is perhaps demonstrated by the fact that localities in which there is agreement across at least three of the vulnerability indexes, and where there may have been a focused effort to increase vaccine coverage, sit at 60–70% vaccine coverage of adults, whereas localities largely recognized as less vulnerable have lower rates of vaccine coverage, some below 50% (see Tables 3, 4). For policy- and decision- making, this presents a problem. While the potential application of vulnerability indexes to target vaccine programs may have resulted in a “higher than the lowest” level of coverage, they have not achieved herd immunity, and have left other localities further behind.

In designing and deploying vaccine distribution schemes, the goal is equitable vaccine uptake that overcomes vulnerability. However, when left to rely on vulnerability indexes that (1) were not built for COVID-19 vaccine uptake and therefore do not include variables addressing multiple dimensions of vaccine uptake, and (2) do not agree with each other, practitioners are left to make decisions with extremely limited knowledge. Thus, given the complex- and context-specific nature of vaccine uptake, this study recommends defining vulnerability specifically for vaccine uptake at the local level, revisiting the definition regularly to critically evaluate and determine any evolutions in the ground truth of vulnerability, and modernizing data collection tools and data analysis for indexes to reflect vulnerability based on these adaptive processes.

This analysis is limited in its use as it assesses only a small number of localities’ COVID-19 vaccine uptake data in comparison with the vulnerability indexes discussed, leaving room for further, more in-depth analysis. This study should thus be expanded using publicly available state data on vaccination doses at the census tract or county level to cross-references these values over time with the vulnerability risk indexes, to determine statistically which index is most appropriate, and to what degree. However, the publicly available data for the Commonwealth is limited in that historic vulnerabilities are only shown separately, meaning any analysis using this data is unable to examine the intersections of vulnerabilities, such as race, sex, age, and locality.

The general disagreement between indexes identified in this analysis demonstrates that vulnerability is a concept which requires a context-specific definition. Further, it demonstrates the way in which vulnerability is characterized quantitatively, from the selection of variables for inclusion to decision-making around weighting and variable importance, may directly affect the agencies’ and policymakers’ understanding not only of what vulnerability is but also of who is vulnerable. The (dis)agreement between these vulnerability indexes, and their inability to detect certain localities that in fact had very low vaccine uptake and should have been considered vulnerable, exemplifies the need to continuously re-examine the definitions of vulnerability used to inform public health decision-making.

Throughout the pandemic, officials have used the best tools available to them. Many have employed the CDC’s SVI, Surgo Venture’s CCVI, or a similar health and well-being focused index (44). As we have explored, however, these measures of social vulnerability may not be appropriate for long-term, sustained vaccine outreach programs, as they were built under different operating definitions of vulnerability than what may apply today, and in the context of the COVID-19 vaccines. In the face of public health crises, and especially in the face of emergent crises, critically assessing how vulnerability is understood, defined, measured, and captured is key. Further, as vulnerability is not a static concept—it evolves—so too should our consideration of vulnerability. Thus, policy- and decision- makers should consider not only what is in a vulnerability index, but why or why are not certain variables—like disability—included and considered in terms of how they intersect across other phenomena that generate vulnerability—gender, race, economic status—in such a way that more accurately represents the lived experience of those facing ‘vulnerability.’

## Data availability statement

Publicly available datasets were analyzed in this study. This data can be found here: https://precisionforcovid.org/ccvi#:~:text=Surgo%20Ventures%20created%20the%20COVID,develop%20solutions%20to%20help%20them, https://www.atsdr.cdc.gov/placeandhealth/svi/index.html#:~:text=Social%20vulnerability%20refers%20to%20the,human%20suffering%20and%20economic%20loss, https://experience.arcgis.com/experience/376770c1113943b6b5f6b58ff1c2fb5c/page/SoVI/, and https://apps.vdh.virginia.gov/omhhe/hoi/dashboards.

## Author contributions

LCS contributed to conversations about the development of the paper, developing the paper structure, constructing the majority of the literature review, and contextualizing the analysis of the data in terms of current and ongoing public health concerns and debates and provided feedback and input on the development and analysis of the results. EF contributed to conversations about the idea of the paper, provided input about the study and paper structure, categorized and evaluated the methodology documents of the four indexes, and summarized findings as maps, tables, and charts, as well as provided feedback and revisions to improve the literature review and discussions. All authors contributed to the article and approved the submitted version.

## Funding

This work was funded by the Hampton Roads Biomedical Research Consortium 2021-22 Collaboration Accelerator Fund (CAF) Grant number: 220118.

## Conflict of interest

The authors declare that the research was conducted in the absence of any commercial or financial relationships that could be construed as a potential conflict of interest.

## Publisher’s note

All claims expressed in this article are solely those of the authors and do not necessarily represent those of their affiliated organizations, or those of the publisher, the editors and the reviewers. Any product that may be evaluated in this article, or claim that may be made by its manufacturer, is not guaranteed or endorsed by the publisher.
