# Supplementary material for: The shortfalls of vulnerability indexes for public health decision-making in the face of emergent crises: the case of COVID-19 vaccine uptake in Virginia
Source: Front Public Health. 2023 May 3;11:1042570. doi: 10.3389/fpubh.2023.1042570 (PMC10188971; doi:10.3389/fpubh.2023.1042570)
Supplement: Supplementary file 1 [file Data_Sheet_1.docx]

**Supplementary Material**

**Table 5. Percentage of Eligible Population Fully Vaccinated in Six Counties that at Least Three Indexes Agreed Were "Vulnerable" – Breakdown by Race**

|  | *Adult* | *Asian or Pacific Islander* | *Black* | *Latino* | *Native American* | *White* |
| --- | --- | --- | --- | --- | --- | --- |
| BRUNSWICK COUNTY | 64.8 | 24.7 | 53 | 95.7 | 39.5 | 50.2 |
| CHARLOTTE COUNTY | 63.3 | 97.4 | 64.6 | 54.5 | 53.7 | 48.6 |
| FRANKLIN COUNTY | 57.3 | 71.8 | 42.8 | 56.7 | 85.8 | 44.1 |
| LEE COUNTY | 46.7 | 26.9 | 5.4 | 14.8 | 38.0 | 42.1 |
| MECKLENBURG COUNTY | 65.5 | 58.4 | 59.0 | 72.9 | 51.6 | 53.1 |
| NOTTOWAY COUNTY | 65.7 | 77.0 | 50.1 | 45.8 | 37.9 | 48.5 |

**Table 6. Percentage of Eligible Population Fully Vaccinated in Six Counties that had Very Low Vaccination Uptake but Were Not Agreed Upon as "Vulnerable" – Breakdown by Race**

|  | *Adult* | *Asian or Pacific Islander* | *Black* | *Latino* | *Native American* | *White* |
| --- | --- | --- | --- | --- | --- | --- |
| PRINCE EDWARD | 49.8 | 46.3 | 50.1 | 37.3 | 75.4 | 40.1 |
| LYNCHBURG | 54.0 | 53.8 | 47.0 | 37.5 | 84.7 | 45.5 |
| CARROLL | 48.0 | 90.2 | 27.6 | 36.6 | 62.3 | 40.3 |
| CRAIG | 46.7 | 26.9 | 5.4 | 14.8 | 38.0 | 42.1 |
| TAZEWELL | 51.8 | 36.4 | 20.7 | 39.8 | 57.6 | 34.3 |
| PATRICK | 51.3 | 72.6 | 45.1 | 42.1 | 42.6 | 43.5 |

**Table 7. Localities Identified by at Least One Vulnerability Index as Being in the Top 20% Most Vulnerable in Virginia**

| **FIPS** | **Locality** | **Surgo CCVI** | **CDC CCI** | **FEMA SOVI** | **VDH HOI** |
| --- | --- | --- | --- | --- | --- |
| 51001 | ACCOMACK COUNTY | 1 | 1 | 0 | 0 |
| 51013 | ARLINGTON COUNTY | 0 | 0 | 0 | 1 |
| 51017 | BATH COUNTY | 0 | 0 | 1 | 0 |
| 51520 | BRISTOL CITY | 1 | 0 | 1 | 0 |
| 51025 | BRUNSWICK COUNTY | 1 | 1 | 0 | 1 |
| 51027 | BUCHANAN COUNTY | 0 | 1 | 0 | 0 |
| 51029 | BUCKINGHAM COUNTY | 0 | 1 | 0 | 1 |
| 51530 | BUENA VISTA CITY | 0 | 0 | 1 | 0 |
| 51033 | CAROLINE COUNTY | 0 | 0 | 0 | 1 |
| 51035 | CARROLL COUNTY | 0 | 0 | 0 | 1 |
| 51036 | CHARLES CITY COUNTY | 0 | 0 | 0 | 1 |
| 51037 | CHARLOTTE COUNTY | 0 | 1 | 1 | 1 |
| 51041 | CHESTERFIELD COUNTY | 0 | 1 | 0 | 1 |
| 51570 | COLONIAL HEIGHTS CITY | 1 | 0 | 1 | 0 |
| 51047 | CULPEPER COUNTY | 0 | 1 | 0 | 1 |
| 51049 | CUMBERLAND COUNTY | 0 | 1 | 0 | 1 |
| 51590 | DANVILLE CITY | 1 | 0 | 1 | 0 |
| 51051 | DICKENSON COUNTY | 0 | 1 | 0 | 1 |
| 51053 | DINWIDDIE COUNTY | 0 | 1 | 0 | 0 |
| 51595 | EMPORIA CITY | 1 | 0 | 1 | 0 |
| 51057 | ESSEX COUNTY | 0 | 1 | 0 | 0 |
| 51063 | FLOYD COUNTY | 0 | 0 | 0 | 1 |
| 51065 | FLUVANNA COUNTY | 0 | 0 | 0 | 1 |
| 51067 | FRANKLIN COUNTY | 0 | 1 | 1 | 1 |
| 51640 | GALAX CITY | 1 | 0 | 1 | 0 |
| 51077 | GRAYSON COUNTY | 0 | 0 | 1 | 0 |
| 51083 | HALIFAX COUNTY | 0 | 0 | 1 | 0 |
| 51660 | HARRISONBURG CITY | 1 | 0 | 1 | 0 |
| 51089 | HENRY COUNTY | 1 | 0 | 0 | 0 |
| 51091 | HIGHLAND COUNTY | 0 | 0 | 1 | 0 |
| 51670 | HOPEWELL CITY | 1 | 0 | 0 | 0 |
| 51097 | KING AND QUEEN COUNTY | 0 | 0 | 0 | 1 |
| 51103 | LANCASTER COUNTY | 0 | 0 | 1 | 0 |
| 51105 | LEE COUNTY | 1 | 1 | 0 | 1 |
| 51111 | LUNENBURG COUNTY | 0 | 1 | 0 | 1 |
| 51680 | LYNCHBURG CITY | 1 | 0 | 1 | 0 |
| 51113 | MADISON COUNTY | 0 | 0 | 0 | 1 |
| 51690 | MARTINSVILLE CITY | 1 | 0 | 1 | 0 |
| 51115 | MATHEWS COUNTY | 0 | 0 | 0 | 1 |
| 51117 | MECKLENBURG COUNTY | 1 | 1 | 1 | 0 |
| 51125 | NELSON COUNTY | 0 | 0 | 1 | 1 |
| 51127 | NEW KENT COUNTY | 0 | 0 | 0 | 1 |
| 51700 | NEWPORT NEWS CITY | 1 | 0 | 0 | 0 |
| 51710 | NORFOLK CITY | 1 | 0 | 0 | 0 |
| 51131 | NORTHAMPTON COUNTY | 0 | 1 | 1 | 0 |
| 51133 | NORTHUMBERLAND COUNTY | 0 | 0 | 1 | 1 |
| 51720 | NORTON CITY | 0 | 0 | 1 | 0 |
| 51135 | NOTTOWAY COUNTY | 1 | 0 | 1 | 1 |
| 51137 | ORANGE COUNTY | 0 | 1 | 0 | 0 |
| 51139 | PAGE COUNTY | 0 | 1 | 0 | 1 |
| 51141 | PATRICK COUNTY | 0 | 1 | 0 | 1 |
| 51730 | PETERSBURG CITY | 1 | 0 | 1 | 0 |
| 51143 | PITTSYLVANIA COUNTY | 0 | 1 | 0 | 0 |
| 51740 | PORTSMOUTH CITY | 1 | 0 | 0 | 0 |
| 51145 | POWHATAN COUNTY | 0 | 1 | 0 | 0 |
| 51147 | PRINCE EDWARD COUNTY | 0 | 1 | 0 | 1 |
| 51760 | RICHMOND CITY | 1 | 0 | 0 | 0 |
| 51159 | RICHMOND COUNTY | 1 | 0 | 1 | 0 |
| 51770 | ROANOKE CITY | 1 | 0 | 0 | 0 |
| 51161 | ROANOKE COUNTY | 0 | 0 | 1 | 0 |
| 51169 | SCOTT COUNTY | 0 | 1 | 0 | 0 |
| 51171 | SHENANDOAH COUNTY | 0 | 1 | 0 | 0 |
| 51173 | SMYTH COUNTY | 1 | 1 | 0 | 0 |
| 51175 | SOUTHAMPTON COUNTY | 0 | 1 | 0 | 1 |
| 51790 | STAUNTON CITY | 1 | 0 | 1 | 0 |
| 51183 | SUSSEX COUNTY | 1 | 0 | 0 | 0 |
| 51820 | WAYNESBORO CITY | 0 | 1 | 1 | 0 |
| 51840 | WINCHESTER CITY | 1 | 0 | 0 | 0 |
| 51195 | WISE COUNTY | 1 | 0 | 0 | 0 |
